# Multisite Semiautomated Clinical Data Repository for Duplication 15q Syndrome: Study Protocol and Early Uses

**DOI:** 10.2196/resprot.7989

**Published:** 2017-10-18

**Authors:** Oluwaseun Jessica Ajayi, Ebony Jeannae Smith, Teeradache Viangteeravat, Eunice Y Huang, Naga Satya V Rao Nagisetty, Nora Urraca, Laina Lusk, Brenda Finucane, Dimitrios Arkilo, Jennifer Young, Shafali Jeste, Ronald Thibert, The Dup15q Alliance, Lawrence T Reiter

**Affiliations:** ^1^ Biomedical Informatics Core Children's Foundation Research Institute Le Bonheur Children's Hospital Memphis, TN United States; ^2^ Department of Pediatrics University of Tennessee Health Science Center Memphis, TN United States; ^3^ Department of Surgery University of Tennessee Health Science Center Memphis, TN United States; ^4^ Pediatric Clinical Research Unit Le Bonheur Children's Hospital Memphis, TN United States; ^5^ Autism and Developmental Medicine Institute Geisinger Health System Lewisburg, PA United States; ^6^ Minnesota Epilepsy Group Children's Hospitals and Clinics of Minnesota St. Paul, MN United States; ^7^ Center for Autism Research and Treatment Department of Neurology University of California, Los Angeles Los Angeles, CA United States; ^8^ Department of Neurology Massachusetts General Hospital Harvard Medical School Boston, MA United States; ^9^ Dup15q Alliance Highland Park, IL United States; ^10^ Department of Neurology University of Tennessee Health Science Center Memphis, TN United States; ^11^ Dup15q Alliance Fayetteville, NY United States

**Keywords:** clinical data repository, Dup15q syndrome, patient-centered, clinical phenotypes, cohort discovery

## Abstract

**Background:**

Chromosome 15q11.2-q13.1 duplication syndrome (Dup15q syndrome) is a rare disorder caused by duplications of chromosome 15q11.2-q13.1, resulting in a wide range of developmental disabilities in affected individuals. The Dup15q Alliance is an organization that provides family support and promotes research to improve the quality of life of patients living with Dup15q syndrome. Because of the low prevalence of this condition, the establishment of a single research repository would have been difficult and more time consuming without collaboration across multiple institutions.

**Objective:**

The goal of this project is to establish a national deidentified database with clinical and survey information on individuals diagnosed with Dup15q syndrome.

**Methods:**

The development of a multiclinic site repository for clinical and survey data on individuals with Dup15q syndrome was initiated and supported by the Dup15q Alliance. Using collaborative workflows, communication protocols, and stakeholder engagement tools, a comprehensive database of patient-centered information was built.

**Results:**

We successfully established a self-report populating, centralized repository for Dup15q syndrome research. This repository also resulted in the development of standardized instruments that can be used for other studies relating to developmental disorders. By standardizing the data collection instruments, it allows us integrate our data with other national databases, such as the National Database for Autism Research. A substantial portion of the data collected from the questionnaires was facilitated through direct engagement of participants and their families. This allowed for a more complete set of information to be collected with a minimal turnaround time.

**Conclusions:**

We developed a repository that can efficiently be mined for shared clinical phenotypes observed at multiple clinic sites and used as a springboard for future clinical and basic research studies.

## Introduction

### Background and Significance

Duplications of the proximal arm of chromosome 15q11.2-q13.1 result in the genetic condition Duplication 15q syndrome (Dup15q syndrome). Duplications can be either interstitial (3 copies) or isodicentric (4 copies). Most cases are *de novo* and maternally derived, although some interstitial paternally derived cases have been described [1]. Common phenotypes in most individuals affected by Dup15q syndrome include hypotonia, motor and language impairment, intellectual disability, autism spectrum disorder, developmental disability, and seizures [2].

The Dup15q Alliance is a nonprofit parent support organization that supports patients and families affected by Dup15q syndrome, organizes yearly scientific and family meetings, and promotes awareness about Dup15q syndrome. The Dup15q Alliance consists of a 12-member Board of Directors, a Professional Advisory Board staffed by clinical and research professionals, several organizational committees, individuals and families living with Dup15q syndrome, and community volunteers [3]. Historically, Dup15q syndrome research has been limited by the relative rarity of this condition and the geographic distance of patients to clinic locations. In an effort to overcome these limitations, the Dup15q Alliance established a group of clinic sites whose goal is to focus specifically on the needs of individuals with this syndrome. As a result of this initiative, a collaboration of 5 Dup15q syndrome clinics has led to the development of a central data repository to support current and future research.

### Objective

The primary objective of this project is to establish a national deidentified database with clinical and survey information on individuals diagnosed with Dup15q syndrome. This paper documents how stakeholder engagement tools, collaboration methodologies, and communication protocols were used to establish a first-of-its-kind national Dup15q syndrome data repository that will support future research efforts and encourage expansion of collaborative research on Dup15q syndrome among academic and medical institutions.

## Methods

### Design

This multisite research collaborative of institutions and individuals with an interest in Dup15q syndrome was initiated with the goal of identifying and developing new treatments and improving the standard of living for individuals with the syndrome and their families. To initiate the project, the Dup15q Alliance organized a series of communication sessions via conference calls and meetings. Attendees included Dup15q syndrome research and clinical professionals from the Dup15q Alliance’s Professional Advisory Board, members of the Dup15q Alliance’s Board of Directors, biomedical informatics professionals, research study coordinators, and other clinical investigation professionals. These communication sessions emphasized the project’s adherence to a team science approach and partnership. Five partnering clinical sites were identified and several key protocols were established, including plans for establishing the necessary compliance and governance for a multisite study of this magnitude, a standardized set of clinical and behavioral metrics to be collected by each site (Table 1), communication protocols to be used for future collaboration among the study participants and administrating partners, technical specifications for the data warehousing tools to collect and store study data, and a stakeholder engagement plan for attracting, engaging, and retaining additional participants and families.

### Setting

The 5 primary Dup15q syndrome clinics serve as the foundational setting for the project. These sites are the Dup15q Center of Excellence at Massachusetts General Hospital, the Geisinger Dup15q Developmental Clinic, the Dup15q Clinic at Minnesota Epilepsy Group, the University of Tennessee Le Bonheur Duplication 15q Clinic (closed July 2017), and the University of California Los Angeles Dup15q Clinic. Many of these sites include affiliated universities, research laboratories, and other vital resources that contribute to the project.

Effective implementation of technology tools is essential to support the research. The Research Electronic Data Capture (REDCap) [4] application is used to build our standardized clinical record database and securely store the supporting data for the study. Each clinic securely logs in to the Le Bonheur Children’s Hospital’s instance of REDCap for data collection. The repository is set up to collect longitudinal data. The frequency of the visits is determined by the clinicians at each clinic. At this time, following the first visit, no additional survey information is collected from the families, but the possibility exists for the initiation of multisite subprojects involving survey data. For example, gastrointestinal and nutrition surveys were initiated at the Memphis site. The data from subsequent visits are collected and entered by the clinicians or coordinators. Other Web-based tools and software aimed at supporting team collaboration and data sharing are used to implement the study’s communication protocols, help eliminate geographical barriers, and promote innovation.

**Table 1:** List of tasks involved in the development of data collection instruments and protocols.

Task	Approach
Monthly collaboration sessions to establish data collection instruments and processes	A series of face-to-face meetings and conference calls that included leaders and representatives from various areas of interests from the Dup15q Alliance: researchers, clinicians, biomedical informaticists, and Dup15q syndrome study participants and their families. The resulting products of these meetings were a standardized set of clinical and behavior metrics to be collected across multiple clinical sites and the initial framework for standardized data collection protocols to ensure consistency and reliability in the collected data.
Identification of validated data collection instruments and protocols and acquisition of proper licensing and permission for use	To benefit as much as possible from professionally validated data collection tools, the group decided to identify and incorporate into this study, where appropriate, previously established and validated clinical data collection instruments. Working with the Dup15q Alliance, study partners initiated contact with vendors of data collection instruments and protocols protected by copyright laws to acquire proper licensing and permission to use these tools for this project.
Curating data for compliance with the National Database for Autism Research	Since Dup15q syndrome is one of the most common identifiable molecular causes of autism, a long-term goal of the study is to include the data collected during this multisite study in the data available via request from the National Database for Autism Research (NDAR). We researched NDAR’s data standards to ensure the collection instruments established for this project stored the appropriate variables to generate a global unique identifier for each individual in accordance with NDAR’s data standards.
Building a standardized clinical research database for Dup15q syndrome	The data collection instruments developed in the 3 previous steps of this process were translated into forms and surveys in a standardized clinical research database by the biomedical informatics professionals serving as the data managers for the study. Extensive functionality testing of these electronic forms, surveys, and the logic embedded within them was conducted by the data managers and clinical research collaborators.
Incorporating the data collection instruments into the clinical workflow	Even though data collection is an essential aspect of conducting meaningful research, we did not want data collection to interfere with clinical processes or negatively impact patient treatment. Thus, the biomedical informaticists worked closely with research coordinators and clinicians to optimize the data collection instruments for seamless integration into the clinical workflow. This involved the biomedical informaticists shadowing the clinicians during patient interaction and iterative development of the data collection instruments to improve the electronic forms for use in the clinical settings and enhanced usability of the surveys for study participants and their families.

### Participants and Stakeholders

Most of the study’s participants and stakeholders are based within the clinical sites, including treating physicians, research scientists, research study coordinators, biomedical informatics professionals, and study participants and their relatives. Members of the Dup15q Alliance are also actively involved in this study, serving as administrative leaders and ambassadors for awareness and providing financial support.

### Protection of Human and Animal Subjects

The individuals in this database have signed an informed consent for each clinic site which gives permission for their deidentified clinical and survey data to be collected in the database repository. Each clinic site is responsible for obtaining institutional review board (IRB) approval for this study at their respective institution.

### Compliance and Governance

The relationships among all stakeholders and data type and data exchange among stakeholders was clarified in initial face-to-face meetings and conference calls between the central leadership of the Dup15q Alliance, the principal investigators of this project, and the biomedical informatics team at the Children’s Foundation Research Institute (CFRI) at Le Bonheur Children’s Hospital (LBCH), a large referral hospital in Memphis, TN.

A project plan was drafted documenting the identity and mission of the Dup15q Alliance, project goals, and the role of the LBCH biomedical informatics team as the data management core. In this document, the business relationship between the Dup15q Alliance and the data management core and the relationship between the Dup15q Alliance and the contributing clinics were defined. The project phases and informatics workflow were also elucidated. Once all parties agreed upon the governance and structure, LBCH’s legal team reviewed the plan. Additional discussions and clarification occurred among the partnering groups, including discussion with legal representation of the 2 major partners, the Dup15q Alliance and LBCH. A business associate agreement was drafted and executed between the Dup15q Alliance and LBCH for the data management and related services that the biomedical informatics core would provide to the Dup15q Alliance, in compliance with the Health Insurance Portability and Accountability Act (HIPAA)–Health Information Technology for Economic and Clinical Health Act (HITECH) Omnibus Final Rule. The legal relationships between the Dup15q Alliance and its partner institutions at various clinic site locations as it pertains to data sharing are managed by the Dup15q Alliance. The deidentified clinical and survey data collected from all sites belong to the Dup15q Alliance as per the business agreement.

### Stakeholder Engagement

All participants and families receiving Dup15q syndrome clinical care at the participating sites are approached for voluntary participation in the deidentified data repository.

Prior to any data transfer to the repository database or participation in any additional studies, informed consent is obtained at each clinic site as per their institutional guidelines and federally accredited IRB or equivalent group within the institution. The decision to opt out from participating in the data repository results in continued excellent standard of care for the patient at the specialty clinic without data being entered into the deidentified dataset. If participants and/or families agree to participate, data obtained during clinical evaluation are entered in the database to facilitate standardized procedures across clinic sites. REDCap is used to generate a clinical report for the patient’s medical record prior to a deidentified version being uploaded to the research repository database to support research cohort discovery. The REDCap database is password protected and available only to appropriate IRB-approved study personnel on the HIPAA-secure institutional networks as required by applicable state and federal statutes and laws.

### Communication and Collaboration Protocols

Communication and collaboration for the project began with face-to-face meetings with representatives from participating stakeholders. During these meetings, the framework for communication protocols and standards was established to ensure routine follow-up and inclusion of collaborators. Subsequent monthly communication via conference calls and emails ensured collaboration among the study participants.

The biomedical informatics professionals serve as data managers for the study and communicate information related to the study’s integrated database to all other study participants. These biomedical informaticists work with other key stakeholders to establish a communication workflow that allows all study collaborators to be informed of new developments and important information regarding study data. On a monthly basis, biomedical informaticists communicate with research coordinators at each of the participating sites, generate and provide monthly status updates and integrated data summaries to the principal investigator, and are actively involved in face-to-face meetings facilitated by the study’s leading clinical site. When a new site is added to the repository, executed IRB and business associate agreements are obtained from the hosting institution, after which data managers initiate contact with the leadership of the new collaborating site, provide standardized data collection instructions, and conduct iterative communication until the new site’s data collection instruments and procedures are consistent with those of existing clinical partner sites.

To communicate with study participants while also keeping other key stakeholders engaged, we incorporated a novel direct feedback mechanism to include stakeholders in the data collection processes. Once an individual is scheduled to come to a clinic site in the network, they are placed in a holding area of the database where basic information is collected and consent for the repository is obtained. Shortly after study participants and their families consent to participating in the study, they receive the first in a series of electronic questionnaires. Families are continuously engaged throughout the project via these self-paced, electronic surveys sent through REDCap via email to collect data about the study participant’s behavior and health details. This is a semiautomated process where 56% is entered manually by the clinicians/coordinators and 44% is completed directly by the families. Table 2 defines the common questionnaires and instruments that all clinics participating in the repository currently collect.

Data from these instruments are submitted directly to the database through a user-friendly Web interface. We have found this method of stakeholder engagement highly successful as a data collection technique based on the rate of completion of these questionnaires. The high level of responsiveness demonstrates the investment families and affected individuals are willing to commit to research aimed at improving treatment and quality of life for those living with Dup15q syndrome.

### Registry-Building Process, Data Collection, and Sharing

It was necessary to build a central patient registry to allow researchers to seamlessly integrate registry information with clinical data management systems in order to facilitate collaboration, cohort research, management of derivative studies, follow-up scheduling, and data quality management. The primary component of the registry is use of metadata management to collect data and synchronize it between clinics. This metadata is derived from common data elements defined by national standards (for example, the National Database for Autism Research [NDAR]) to ensure interoperability and sharing and provide functions to standardize, add, modify, and configure registry and associated clinic-specific related metadata. The cohort research registry database is a collection of deidentified patient information from the repository data formatted into the traditional data mart architecture with integrated analytical tools to search for the patient populations that meet certain criteria. This research repository database is integrated with the central registry database to form follow-up research studies based on this unique cohort. Our central registry maintains data integrity and data quality over its entire life cycle, which includes storing, processing, and retrieving data. The registry provides researchers with tools for creating additional data collection instruments, security control, follow-up scheduling, auditing for missed follow-ups, and quality management on the data collected to identify discrepancies or missing information for the data collection.

**Table 2:** List of standardized questionnaires and instruments.

Standard collection tools	Description	Who completes?
Duplication 15q screening questionnaire	This questionnaire collects information about the child’s name and the child’s eating habits, sleeping habits, seizures, and medications being taken. Its purpose is to act as a guide to determine which specialties the child needs to see when coming to the clinic.	Parent
Demographics	Race, sex, and ethnicity of the child, birth mother, and birth father based on the Clinical Data Acquisition Standards Harmonization standard.	Parent
Consent and Duplicaton 15q subtype	This instrument is used to document if the patient’s family consented to be a part of the database. It also is used to document the duplication subtype of the patient for categorization.	Clinician/ coordinator
Seizures	This questionnaire collects information on participants who have and/or are experiencing seizures. It documents types of seizures, medications the patient has taken and side effects, and the age of the participant when the seizures presented.	Parent
General physical and neurological exam	This instrument documents any body system abnormalities that may be observed at the time of the examination. This includes measures such as cerebellar/coordination, muscle strength, plantar response, reflexes, and muscle tone and bulk.	Clinician/ coordinator
Children’s Sleep Habits Questionnaire (CSHQ) [5]	The CSHQ is a parent/guardian-reported screening questionnaire used to document child’s typical sleep habits, sleep behavior, and any difficulties they may have regarding sleeping and waking.	Parent
Family history	This questionnaire is used to document the conditions prevalent in the child’s family history. Additionally, it also collects the history of related information from pregnancy through birth.	Parent
Autism Diagnostic Observation Schedule (ADOS) [6]	The ADOS is a semistructured assessment of communication, social interaction, and play (or imaginative use of materials) for individuals suspected of having autism or other pervasive developmental disorders.	Clinician/ coordinator
Mullen Scales of Early Learning [7]	The Mullen Scales of Early Learning serve the purpose of assessing cognitive and motor ability. Five scales—gross motor, visual reception, fine motor, expressive language, and receptive language—are used for targeting strengths and weaknesses in children. The Mullen test is generally used for evaluating intellectual development and readiness for school.	Clinician/ coordinator
Questionnaire of pediatric gastrointestinal symptoms	This questionnaire documents information about the child’s reflux issues and bowel movements (gastrointestinal problems are often coincident with autism).	Parent
Vineland Adaptive Behavior Scales II [8]	The Vineland II is an instrument used for diagnosing and classifying intellectual and developmental disabilities and other disorders such as autism, Asperger syndrome, and developmental delays.	Clinician/ coordinator
Seizures in Dup15q	This seizure questionnaire is a more comprehensive survey about seizures participants may be experiencing.	Parent
Child Behavioral Checklist (CBCL) [9]	The CBCL is a parent-reported questionnaire that documents where the child was rated on various behavioral and emotional problems.	Clinician/ coordinator
Social Communication Questionnaire (SCQ) [10]	The SCQ helps evaluate communication skills and social functioning in children who may have autism or autism spectrum disorders.	Clinician/ coordinator
Social Responsiveness Scale (SRS-2) Scores [11]	The SRS-2 is used to identify social impairment associated with autism spectrum disorders and quantifies its severity.	Clinician/ coordinator
Behavioral Assessment System for Children (BASC-2) Scores [12]	The BASC-2 tool is used to evaluate changes in behavior or emotional status.	Clinician/ coordinator

**Figure 1:**
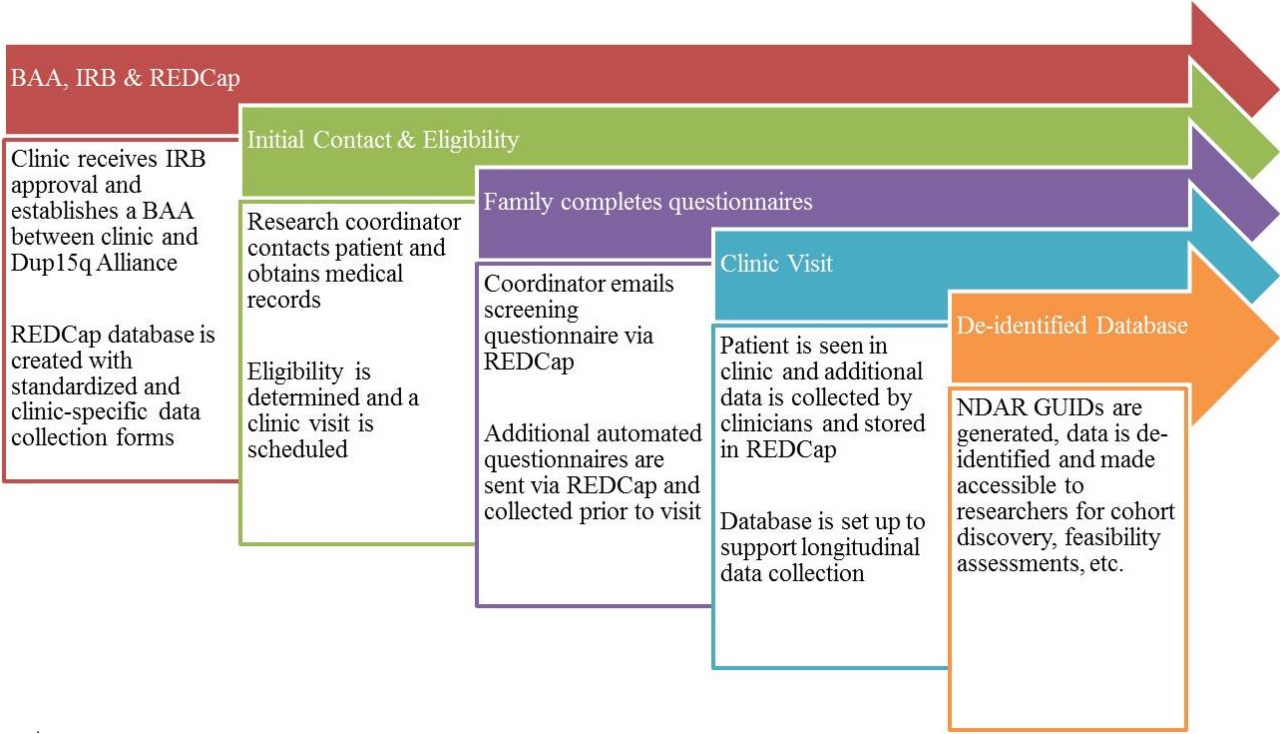
Schematic of workflow process.

Only deidentified aggregate data is available to researchers from the central registry via the research cohort repository database. Clinical data can be disseminated from the repository database as a limited data set in order to gain insights into the condition, with the end goal being ongoing improvement of patient care. Research data use agreements and local institution IRB approval must be signed by a researcher before a limited data set can be shared. The Dup15q Alliance and LBCH data management core are working together to develop policies and procedures addressing a process flow to handle requests for access to the data from nonparticipant stakeholders. It is our goal that meaningful results will be presented at local, regional, national, and international meetings as well as published in peer-reviewed journals and professional newsletters and, eventually, included in textbooks. Figure 1 illustrates the overall workflow of this process. The consortium provides semiannual summaries of research activities, including comprehensive presentation and publication lists, to participants through the Dup15q Alliance website and mass emails. Because this is an ongoing clinical endeavor and a repository database, it is unclear when the clinical repository data collection phase will end.

## Results

**Figure 2:**
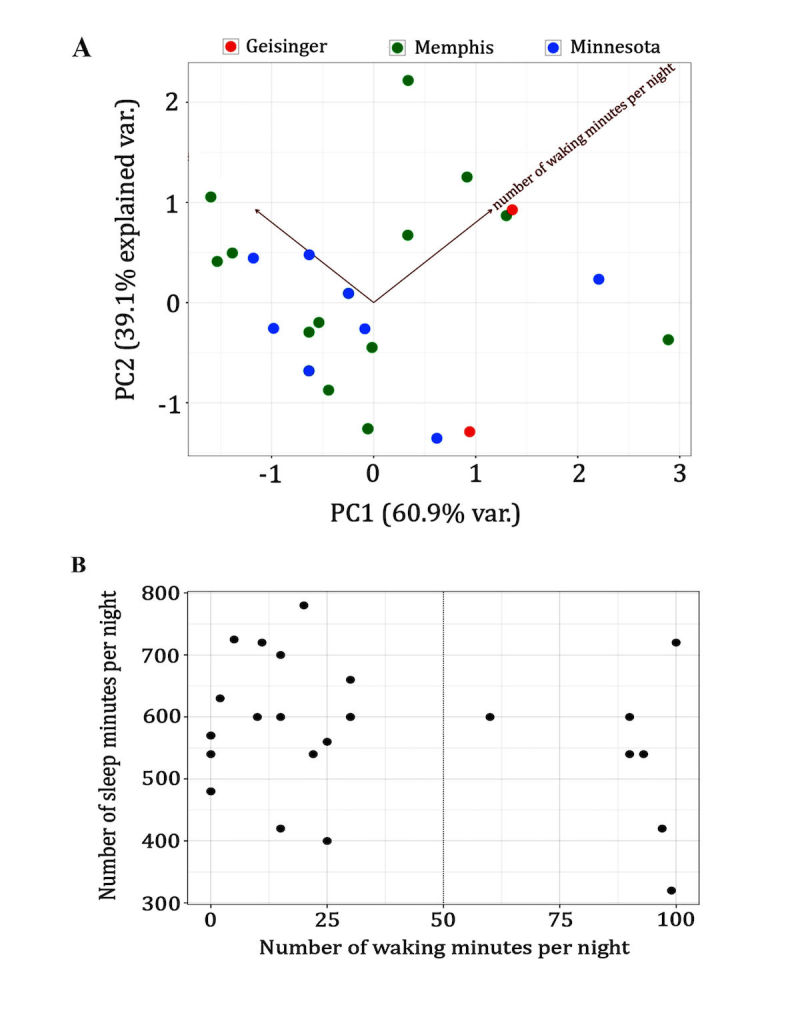
(A) A plot of 2 principal components (minutes per night vs number of waking minutes per night) applied to patient sleep date (N=23) from 3 sites. Note that there is a clear separation between groups of data but that both groups contain individuals from all 3 sites. (B) Plot of the number of sleep minutes versus the number of waking minutes (N=23). Note that 7/23 individuals were awake >50 minutes once aroused, while the majority of subjects stayed awake less than 25 minutes.

The Dup15q repository was built in 2014 by the CFRI biomedical informatics team at the University of Tennessee Health Science Center Le Bonheur Duplication 15q clinic in Memphis, Tennessee, under the direction of Dr Lawrence Reiter. It currently houses data from 96 participants with Dup15q syndrome. Of the participants with demographic data captured, 9.7% were African American, 85.5% were white, <2% were Asian, 45.2% were male, and 79.1% were between 2 and 16 years old (mean age 7.56 [SD 6.02] years [range 1 to 26 years]). Much of the collected data (deidentified) capture features of the condition such as intellectual, motor, and behavioral difficulties; speech and language problems; seizures; and sleep disturbances and gastrointestinal problems. It allows us to begin a dialog about the patterns emerging from these data sets. For example, we found evidence for 2 types of sleep disturbances in people with Dup15q syndrome. In both, individuals awaken in the middle of the night. However, most of these individuals fall back to sleep in under 25 minutes, whereas a small number remain awake for more than an hour (Figure 2). Furthermore, principal component analysis [13] reveals that the driving factor behind this variance is not where the individual’s data were collected, meaning that this is a sleep issue across individuals at multiple clinic sites. The repository will soon be used to study the effectiveness of various medications such as those for epilepsy in people with Dup15q syndrome.

## Discussion

### Initial Developments and Findings

This repository created a collection of standardized instruments that are used across all clinic sites for collecting data. Because these instruments were derived from common data elements defined by national standards, such as the NDAR, these tools are directly transferrable to other studies involving developmental disorders or autism spectrum disorders.

One aspect that sets this multisite study apart from other similar research projects is the reliance on study participants and their families for direct data entry and curation of study data. We found that families were very compliant and completed 82.4% of the questionnaires assigned to them. Typically, very few families needed follow-up reminders before completing the electronic surveys that were sent to participants and their families. The turnaround time for questionnaires being sent to survey completion is minimal. This may be a reflection of how vested these individuals are in participating in studies that can help reach the goals of this research alliance.

### Challenges

One of the most challenging aspects of the project was establishing a common dataset to be collected across all participating clinical sites. It was important to limit the information being collected to a reasonable number of data elements that can be entered consistently by various data curators while also collecting enough data to allow for meaningful future analysis to support a multitude of yet-to-be-determined research hypotheses.

The number of clinical partners participating in the study had both positive and negative effects. Increasing the number of clinical sites involved in the study allowed for a more diversified population of Dup15q syndrome study participants and a higher volume of patient data collected throughout the life of the study. However, increased number of clinical partners also meant having a larger number of institutions with varying areas of interests, all of whom had to agree on the data points to be collected and the governance model that prescribed how those data are to be managed and used. Understandably, each site has their own vested interests, organizational policies, and experienced clinical research leaders with differing opinions on how to accomplish this goal.

Through monthly collaboration meetings with researchers, physicians, and family representatives, the group established a well-defined scope for this project that met many of the common research objectives of all participating clinical partners. To address research objectives beyond the scope of this project, the group established a compromise that allowed each clinical site to collect, in addition to the common dataset, additional data to support each site’s institutional research objectives and strengths in areas such as epilepsy, developmental disorders, genetics, and autism.

We found it challenging for data to be consistently entered by the clinicians. While we optimized the data collection instruments for seamless integration into the clinic’s workflow, in some cases, these instruments could not be completed online. This then required clinicians to expend time to go back and enter data manually. This proved to be more challenging than originally expected. Also, in some cases, moving from paper to electronic forms was not an option for some of the instruments used in the repository. This challenge was addressed by integrating reminders for the clinicians into our communication plan to encourage timely data entry. We also followed up with individual clinicians, if needed.

### Conclusion

The successful initialization of this centralized clinical data repository provides a resource for clinicians, researchers, research subjects, and others affected by or interested in Dup15q syndrome to develop a better understanding of this rare condition through combined data from many clinic sites. Our future work includes the development of a cohort discovery platform that will allow researchers to engage with the Dup 15q Alliance, increase the number of collaborations across clinical and academic institutions, and improve the overall treatment and quality of care for those living with Dup15q syndrome.

## Abbreviations

ADOS: Autism Diagnostic Observation Schedule
BASC-2: Behavioral Assessment System for Children
CBCL: Child Behavioral Checklist
CFRI: Children’s Foundation Research Institute
CSHQ: Child Sleep Habits Questionnaire
Dup15q syndrome: Duplication 15q syndrome
HITECH: Health Information Technology for Economic and Clinical Health
HIPAA: Health Insurance Portability and Accountability Act
IRB: institutional review board
LBCH: Le Bonheur Children’s Hospital
NDAR: National Database for Autism Research
REDCap: Research Electronic Data Capture
SCQ: Social Communication Questionnaire
SRS-2: Social Responsiveness Scale
